# Gastric Staple Line Reinforcement With Ligation Clips for Hemostasis in Laparoscopic Sleeve Gastrectomy

**DOI:** 10.7759/cureus.37271

**Published:** 2023-04-07

**Authors:** Muhammad Saulat Naeem, Rooh ul Ain, Zoya Sadiq, Aniqa Ejaz, Usama Rafi, Muhammad Awais, Maaz Ul Hassan

**Affiliations:** 1 Surgery, Mayo Hospital, Lahore, PAK; 2 Surgery, Shalamar Medical and Dental College, Lahore, PAK; 3 Medicine, Naeem Hospital, Gujranwala, PAK; 4 Surgery, Tipperary University Hospital, Clonmel, IRL

**Keywords:** stapled anastomosis, difficult hemostasis, minimal access bariatric and laparoscopic surgery, liga clips, hemostatic clip, gastric sleeve surgery, gastric sleeve

## Abstract

Introduction: In Pakistan and worldwide, there is a lack of literature on the role of ligation clips in controlling hemostasis in laparoscopic sleeve gastrectomy. ligation clips are being used to secure hemostasis and act as staple line reinforcement to prevent intra-operative and postoperative bleeding. Data can be utilized to reflect the role of staple line reinforcement with ligation clips so as to guide surgeons about its safety and efficacy.

Methodology: This retrospective observational study was conducted at the Shalamar Hospital, Lahore, Pakistan, and included 120 patients. The patients’ demographic parameters, BMI, intra-operative and postoperative bleeding in terms of hematemesis, melena, and fall in hemoglobin (Hb) postoperatively were compared to preoperative Hb and recorded.

Result: One hundred and twenty cases of laparoscopic sleeve gastrectomy (LSG) were included with a mean age of 44.77±12.05 years. The mean BMI was 52.06±13.85 preoperatively. The mean drop in hemoglobin was 0.04±0.26 with a p-value of 0.07, which is statistically insignificant. Among 120 cases, two (1.7%) patients reported episodes of melena, and four (3.3%) patients reported hematemesis. Postoperative hypotension was recorded in six (5%) patients and eight (6.7%) patients had postoperative tachycardia.

Conclusion: This study shows ligation clip application along the staple line is an effective means of hemostasis similar to suture application.

## Introduction

Laparoscopic sleeve gastrectomy (LSG) is widely used for the surgical management of morbid obesity and is the most commonly performed bariatric procedure [1]. Weight loss may be achieved by gastric resection and malabsorption and also by change in hormonal secretion. Originally sleeve gastrectomy was only the initial procedure in patients with BMI>50 to reduce weight to safer levels to perform more complex procedures such as Roux en Y gastric bypass or biliopancreatic diversion. But overwhelming results in terms of weight reduction and improvement in comorbidities lead to the adoption of sleeve gastrectomy as a primary bariatric procedure [2]. Short-term studies show that the LSG is safer than Roux-en-Y gastric bypass with short procedure time and hospital stay [3].

Despite having improved morbidity and mortality, LSG still carries some risks. Hemorrhage is one of the most common complications of this surgery with an incidence of 4.94% [4]. It can lead to revision surgery and delay the healing of anastomosis, which in turn may cause an anastomotic leak and increase the risk of infection/abscess [5].

Postoperative bleeding can be classified on the basis of the site of bleeding into intra-luminal and intra-abdominal bleeding [6-8]. The former type presents as hematemesis or melena in addition to hypotension and tachycardia and is usually the result of staple line failure with an incidence of 1-3% [9] and can be prevented by staple line reinforcement [10,11], which can be achieved by over-sewing the staple line, omental wrap, or fibrin glue [12]. However, the intra-abdominal bleeding site can be at the staple line as well as other sources such as omentum, spleen, liver, or abdominal wall at the site of trocar entry [13], and is indicated by blood in the abdominal drain [14] along with hypotension and tachycardia.

A randomized trial concluded that routine elevation of systolic blood pressure to 140mmHg near the end of the procedure to identify bleeding points and over-suture of the staple line can decrease hemorrhagic complications with a reasonable increase in procedure time [15]. In routine, it is common practice to apply ligation clips to all suspected bleeding points during the procedure to avoid hemorrhagic complications.

These surgical ligation clips were invented by Ernest C. Wood in 1968 [16]. They work by approximation of tissues to one another and are most beneficial when used over tubular structures such as blood vessels. The use of ligation clips for the ligation of cystic artery and duct during laparoscopic cholecystectomy has been widely studied and found safe and effective [17,18]. However, limited publications are available regarding their efficacy in the control of hemostasis during LSG. Most studies have been focused on staple line leaks but the prevention of staple line bleeding and its control has suffered neglect in literature.

Worldwide, there is a lack of literature on the role of ligation clips in controlling hemostasis in LSG. Bariatric procedures are performed in high numbers at Shalamar Hospital, Lahore, Pakistan, and ligation clips are being used to secure hemostasis and act as staple line reinforcement to prevent intra-operative and postoperative bleeding. In this study, the data were analyzed to reflect the role of staple line reinforcement with ligation clips to guide surgeons regarding its safety and efficacy.

## Materials and methods

One hundred and twenty patients who had undergone laparoscopic sleeve gastrectomy at Shalamar Hospital, Lahore, Pakistan, between 2017 and 2021 were identified using the hospital database system retrospectively. All surgeries were performed by a single surgeon and the same surgical team with more than five years of experience in this field. All patients with bleeding disorders and patients needing re-exploration for bleeding other than staple line were excluded. After informed consent, online and paper-based records were reviewed for specifics of data. The patients' demographic parameters, BMI, intra-operative and postoperative bleeding in terms of hematemesis, melena, and fall in hemoglobin postoperative as compared to preoperative Hb were compared and recorded. A hemoglobin loss of >2g/dl is considered significant. Patients' re-admission data if any was obtained, which included any further hospital visits for postoperative bleeding up to one month following surgery.

Surgical technique

Preoperatively, patients were given low molecular weight heparin, fluid bolus, and dexamethasone according to weight. Intraoperatively patients were kept in a supine, reverse Trendelenburg position. The standard ports technique for LGS was utilized and pneumoperitoneum was created via the Visiport technique. The omentum was separated from the greater curvature of the stomach using LigaSure™ vessel sealing device (Medtronic plc, Dublin, Ireland) starting at the level of crow's foot above up to the angle of His and below till the level of the pylorus, stomach freed posteriorly. A 32 Fr bougie was inserted and the stomach was dissected along this while using an Echelon 60mm Endo stapling gun (Ethicon, Inc., Raritan, New Jersey, United States) starting 3-4 cm from the pylorus up to the angle of His. Hemostasis secured along the staple line with LIGACLIP® LT300 (Ethicon Inc.) along the obvious spurting vessels. Gastric leak was checked via inflation of gastric remnant with 60 ml of methylene blue. Resected stomach was sent for histopathology. Postoperatively, for thromboembolic prophylaxis, thromboembolic deterrent (TED) stockings and low molecular weight heparin were given for four weeks. IV fluid was given for a week and oral liquid was started after 12 hours postoperatively.

Statistical analysis

All data were analyzed by using IBM SPSS Statistics for Windows, Version 23.0 (Released 2015; IBM Corp., Armonk, New York, United States). Quantitative variables were presented as mean ± standard deviation. Categorical variables were presented as frequencies percentage. A paired t-test was used to compare the last follow-up point and baseline at the time of surgery. The determining factors for hemostasis control were noted and analyzed, and a comparison was done using log-rank method. A p-value <0.05 was considered statistically significant.

## Results

A total of 120 patients undergoing LSG were enrolled in this study. The mean age of patients was 44.77±12.05 years (range 19-69 years). This included 39 (32.5%) males and 81 (67.5%) females.

Preoperative assessment, height, weight, and BMI of each patient were noted from the records. The mean BMI was 52.06±13.85 (range 32.4-120.3). The hemoglobin value of each patient was noted both preoperatively and postoperatively from the patient’s records and the difference in their value was determined. The mean baseline hemoglobin of our patients was 12.64g/dl±1.27 (range 9.7-15.4) and the mean postoperative hemoglobin was 12.59g/dl±1.29 (range 9.7-15.4). The mean difference between the two values was 0.04±0.26. No significant postoperative hemoglobin drop was noted and the maximum hemoglobin drop was 1.9, which was not full-filling hemoglobin drop criteria (Table 1).

**Table 1:** Variables with baseline and postoperative hemoglobin

Variables	Mean ± SD
Age	44.77±12.05 years
BMI	52.06±13.85
Baseline hemoglobin	12.64g/dl±1.27
Postoperative hemoglobin	12.59g/dl±1.29
Difference between preoperative and postoperative hemoglobin	0.04±0.26.

Among 120 cases, two (1.7%) patients reported episodes of melena, and four (3.3%) patients reported hematemesis. Postoperative hypotension was recorded in six (5%) patients. Eight (6.7%) patients had postoperative tachycardia (Table 2).

**Table 2:** Frequency distribution among melena, hematemesis, postoperative hypotension, and postoperative tachycardia

Variables	Frequency (%)
Melena	2 (1.7%)
Hematemesis	4 (3.3%)
Postoperative hypotension	6 (5%)
Postoperative tachycardia	8 (6.7%)

Postoperative tachycardia and hypotension were compared with melena and hematemesis and it was found that eight patients had postoperative tachycardia, of which two patients had melena, which was statistically significant (p-value: 0.001). Six patients had postoperative hypotension, of which two patients had melena, which was also statically significant (p-value: 0.001) (Table 3). 

**Table 3:** Frequency percentage of melena against postoperative tachycardia and hypotension

			Melena		Total	
			Yes	No		
Postoperative Tachycardia	Yes	Count	2	6	8	
	No	Count	0	112	112	
Total		Count	2	118	120	
		% of Total	1.7%	98.3%	100%	
Postoperative Hypotension	Yes	Count	2	4	6	
	No	Count	0	114	114	
Total		Count	2	118	120	
		% of Total	1.7%	98.3%	100%	

Eight patients had postoperative tachycardia out of which four patients had hematemesis, which was statistically significant (p-value: 0.001). Six patients had postoperative hypotension, of which two patients had hematemesis, which was also statically significant (p-value: 0.001) (Table 4).

**Table 4:** Frequency percentage of hematemesis against postoperative tachycardia and hypotension

					Hematemesis		Total	
					Yes	No		
Postoperative Tachycardia		Yes	Count		4	4	8	
		No	Count		0	112	112	
Total			Count		4	116	120	
			% of Total		3.3%	96.7%	100.0%	
Postoperative Hypotension	Yes			Count	2	4	6	
	No			Count	2	112	114	
Total				Count	4	116	120	
				% of Total	3.3%	96.7%	100.0%	

In subset analysis, eight patients had postoperative tachycardia, two patients had hematemesis, and two patients had both hematemesis and melena; this was statistically significant with a p-value of 0.001. Six patients had postoperative hypotension, two had both hematemesis and melena, and this was also statistically significant with a p-value of 0.004 (Table 5).

**Table 5:** Frequency percentage of hematemesis and hematemesis and melena against postoperative tachycardia and hypotension

				Hematemesis	Hematemesis and Melena	Nil	Total
Postoperative Tachycardia	Yes	Count		2	2	4	8
	No	Count		0	0	112	112
Total		Count		2	2	116	120
		% of Total		1.7%	1.7%	96.7%	100%
Postoperative Hypotension	Yes		Count	0	2	4	6
	No		Count	2	0	112	114
Total			Count	2	2	116	120
			% of Total	1.7%	1.7%	96.7%	100%

## Discussion

While other procedures like biliopancreatic bypass and mini gastric bypass and their variants are also practiced frequently depending on the surgeon’s choice, expertise, and their indications, LSG is the most common bariatric procedure worldwide. As with any surgical procedure, certain complications are expected, the common complication because of its long staple line is leakage and bleeding from the staple line. It increases the average cost and operative time [7]. The average incidence of bleeding in LSG is 4.02% [9]. While the study by D’Ugo and Taha demonstrates much higher bleeding incidence without reinforcement to staple line (9-13.7%) as compared to oversewing (1.4-2%) [6], Siddiq et al.'s study shows no difference in terms of bleeding with both suturing or not suturing the staple line [11].

Limited data is available worldwide to document confidently the efficacy and potential of ligation clips for hemostasis in LSG while many studies have been focused on comparing staple line leaks with and without reinforcement steps.

There are various methods for reinforcement of the staple line to control bleeding and prevent leakage. Commonly used methods are suture reinforcement, bovine precordium, and fibrin glue. Ligation clips were used in this study to prevent intraabdominal staple line bleed. While other methods are costly and time-consuming with increased average operative time, the continuous suture method puts insufficient pressure on the vessel while the hybrid suture technique decreases the bleeding (1.3% vs 0) but increases operative time (48.7 vs 49.5 minutes) [10]. In our study, the average operative time was 41.4 minutes. Hence, the use of ligation clips can be beneficial to secure precious operative and anesthesia time and also the clips can be used wisely and swiftly on vessel spurt to avoid major hemorrhage and panic among the operating surgeon. While considering cost-effectiveness, ligation clips can be used cautiously only for visible bleeds or generously throughout the staple line to secure hemostasis and provide reinforcement and the surgeon’s satisfaction. 

In the study conducted by Khoursheed, 2% of the patients had bleeding in the stapler line supported with sutures, while 0.5% required reoperation. However, the postoperative mortality rate was zero [12]. In the present study, staple line intraluminal bleed was encountered in terms of hematemesis in four (3.3%) and melena in two (1.7%) patients. As per institutional policy, a hemoglobin drop of more than 2 g/dl was determined as the standard while comparing the hemoglobin drop in patients after surgery, while in this study we encountered a drop in hemoglobin of 0.04±0.26, with a p-value of 0.07, which is not significant. There was no patient requiring reoperation and no mortality. In our study, three patients had bleeding incidents from the staple line, which was managed conservatively.

We concluded that hemostasis secure with ligature clips along the staple line was as effective as any other method with less time consumption. Hence, ligation clip utilization can be safely achieved when the operating surgeon has limitations with suturing techniques and/or other methods for control of bleeding that are not readily available or expensive. 

The limitations of this study include the fact that it is a retrospective analysis and has a limited number of patients. As the study excluded patients with bleeding disorders and a single brand, Endo stapling device, was used in all patients, standardization of results for all patient types could be difficult. All the patients received preoperative and postoperative low molecular weight heparin as a set protocol for deep venous thrombosis (DVT) prophylaxis, which could be the basis of minor bleeding in subsets of patients with hematemesis and melena.

Bariatric surgery has emerged as a promising discipline for patients suffering from obesity and needing metabolic surgery for comorbid conditions. This article reflects and addresses a major concern in bariatric surgery and many more areas of interest and queries need further research work and standard protocols set.

## Conclusions

Hemostasis control in any surgical procedure, particularly bariatric surgeries for obese patients, where morbidity and mortality rates could be higher, is of prime importance and certain methods have been devised for this aim. Many methods including ligation clips, sutures, and glues have been used with success. This study shows that ligation clip application along the staple line is an effective means of hemostasis similar to suture application.

Hence, ligation clips can be used effectively and swifter than suturing of bleeding sites in LSG to reduce anesthesia time and quicker recovery of the patients as its use is also less troublesome and less frustrating for the surgeon than laparoscopic suturing.
